# High level xylitol production by *Pichia fermentans* using non-detoxified xylose-rich sugarcane bagasse and olive pits hydrolysates

**DOI:** 10.1016/j.biortech.2021.126005

**Published:** 2021-12

**Authors:** Vivek Narisetty, Eulogio Castro, Sumit Durgapal, Frederic Coulon, Samuel Jacob, Dinesh Kumar, Mukesh Kumar Awasthi, Kamal Kishore Pant, Binod Parameswaran, Vinod Kumar

**Affiliations:** a School of Water, Energy and Environment, Cranfield University, Cranfield MK43 0AL, UK; b Department of Chemical, Environmental and Materials Engineering, Universidad de Jaén, Campus Las Lagunillas, 23071 Jaén, Spain; c Department of Pharmaceutical Sciences, Kumaun University, Bhimtal, Nainital 263136, Uttarakhand, India; d Department of Biotechnology, School of Bioengineering, College of Engineering and Technology, Faculty of Engineering and Technology, SRM Institute of Science and Technology, SRM Nagar, Kattankulathur, Chengalpattu District, Tamil Nadu, 603203, India; e School of Bioengineering & Food Technology, Shoolini University of Biotechnology and Management Sciences, Solan 173229, Himachal Pradesh, India; f College of Natural Resources and Environment, Northwest A&F University, Yangling, Shaanxi Province 712100, China; g Department of Chemical Engineering, Indian Institute of Technology Delhi, Hauz Khas, New Delhi 110016, India; h Microbial Processes and Technology Division, CSIR-National Institute for Interdisciplinary Science and Technology (CSIR-NIIST), Thiruvananthapuram, Kerala 695019, India

**Keywords:** Xylose, Xylitol, *Pichia fermentans*, Sugarcane bagasse, Olive pits

## Abstract

Hemicellulosic sugars, the overlooked fraction of lignocellulosic residues can serve as potential and cost-effective raw material that can be exploited for xylitol production. Xylitol is a top platform chemical with applications in food and pharmaceutical industries. Sugarcane bagasse (SCB) and olive pits (OP) are the major waste streams from sugar and olive oil industries, respectively. The current study evaluated the potential of *Pichia fermentans* for manufacturing of xylitol from SCB and OP hydrolysates through co-fermentation strategy. The highest xylitol accumulation was noticed with a glucose and xylose ratio of 1:10 followed by feeding with xylose alone. The fed-batch cultivation using pure xylose, SCB, and OP hydrolysates, resulted in xylitol accumulation of 102.5, 86.6 and 71.9 g/L with conversion yield of 0.78, 0.75 and 0.74 g/g, respectively. The non-pathogenic behaviour and ability to accumulate high xylitol levels from agro-industrial residues demonstrates the potential of *P. fermentans* as microbial cell factory.

## Highlights

• Xylitol production from non-detoxified agro-industrial residues using *P. fermentans*.
• Optimum glucose to xylose ratio and feed composition improved Xylitol accumulation.
• Xylitol titer of 86.6 g/L with yield of 0.75 g/g was achieved from SCB hydrolysate.
• OP hydrolysate resulted in xylitol titers of 71.9 g/L with a yield of 0.74 g/g.

## Graphical abstract

**Figure:**
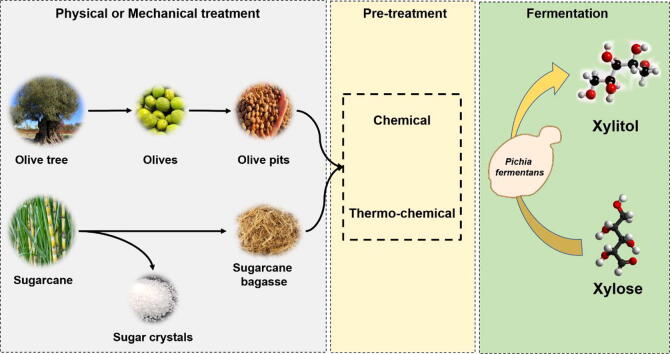

## 1 Introduction

Sugar is an inextricable part of the diet, but the excessive consumption leads to obesity, type II diabetes, and other cardiovascular disorders. Recent research progress on dietary supplementation, lifestyle, and metabolic behaviour, provides us an understanding to transition from health to disease. Therefore, growing awareness on health and fitness, consumers are mostly steered towards healthy foods with low glycemic index rather than unhealthy foods with high sugar levels (Tandel, 2011). The US department of Agriculture recommends 32 g or 8 teaspoons table sugar per day, however, the sugar consumption has been increased to ∼ 150 g/day mostly in children and adolescents. As excessive consumption has negative impact on the health, the medical professionals recommend the non-nutritive or non-sugar sweeteners. Sugars or sweeteners are of two types, nutritive and non-nutritive; nutritive offers high energy as they contain carbohydrates, whereas non-nutritive provides small or no energy (Nayak et al., 2014). Xylitol, a C5 sugar alcohol is one such alternative sweetener with similar sweetness as table sugar but has lower calorific value (1/3^rd^ of table sugar). The US Department of Energy recognised xylitol as the one of the top 12 biobased platform chemicals. A German chemist, Emil Fischer first discovered xylitol in 1891, and since 1960s, it has been supplemented as a sucrose alternative in food, beverage, nutraceutical, and pharmaceutical industries (Cronin, 2003, Tiwari and Baghela, 2020). Xylitol has several health benefits due to its low energy content and anti-carcinogenic properties and can be used in treatment of various diseases like diabetes, colon diseases, haemolytic anaemia, respiratory infections, osteoporosis, iron deficiency, cystic fibrosis, and other pathological disorders (López-Linares et al., 2018, Tiwari and Baghela, 2020). Owing to its inherent nutritional value, and unprecedented demand from various sectors, the global xylitol market exceeded $880 million in 2019, and is expected to grow up to $1.37 billion by 2025 with a Compound Annual growth Rate (CAGR) of 6.6%. The key players of the global market are DuPont, Cargill; CSPC Shengxue Glucose Co., Ltd.; Mitsubishi Shoji Foodtech Co. Ltd.; Novagreen Inc.; Roquette Freres; S2G Biochem; Xylitol Canada, Inc.; Zuchem Inc., and others Xylitol Market Size Worth $1.37 Billion By 2025 | Growth Rate: 6.6% (grandviewresearch.com) (Accessed 22nd September 2021).

Commercially, xylitol is manufactured through chemical route via catalytic Raney Nickel/Al_2_O_3_ mediated hydrogenation of ultrapure D-xylose (xylose + H_2_ → xylitol) at high temperature (150–200 °C) and pressure (50–60 bar) (Tiwari and Baghela, 2020). The process suffers from requirement of ultrapure D-xylose, low catalyst selectivity, low yield (50–60%) and by-product formation with negative environmental impact. Further, both xylose and xylitol, require laborious and expensive separation and purification steps for achieving high level purity and enhance the complexity of the process (Zhang et al., 2021). Whereas, the microbial biosynthesis of xylitol from xylose takes place under mild and environmentally friendly conditions and can utilize crude sugars obtained from the agricultural residues as the carbon source. The biological route for xylitol production using xylose from the hemicellulosic fraction of lignocellulosic feedstocks has emerged as potential alternative which can overcome the disadvantages presented by the chemical route. Lignocellulosic biomass (LCB) is the most abundant renewable feedstock available on this planet. Cellulose, a polysaccharide of β-linked glucose subunits, while hemicellulose, a heteropolysaccharide containing a mixture C5 and C6 sugars with xylose as the most abundant sugar. Xylose is considered inferior in comparison to glucose and hence the hemicellulosic fraction obtained is often discarded or incinerated for power generation. The surplus xylose fraction is overlooked due to lack of xylose utilizing microbial cell factories and suppressed xylose metabolism in the presence of glucose. For example, *Saccharomyces cerevisiae* which is considered as workhorse of modern biotechnology cannot metabolize xylose (Bengtsson et al., 2009). The microbial strains with efficient xylose assimilation and product formation remain a challenge. However, in the last few decades, significant efforts have been made in developing xylose-based cell factories using metabolic and bioprocess engineering approaches. As a result of it, xylose is emerging as a promising substrate for microbial biotechnology as reported elsewhere in the literature (Kwak et al., 2019).

The current study was undertaken for xylitol production by *Pichia fermentans*, a yeast strain isolated by our group. In our previous work, we have shown the potential of *P. fermentans* to manufacture xylitol from pure and xylose-rich crude agricultural residues (Prabhu et al., 2020a). In the present work, xylose was extracted from two abundantly available lignocellulosic feedstocks, sugarcane bagasse (SCB) and olive pits (OP) using an acid-based pretreatment. Glucose was used as a co-substrate to support the cell growth, provide supply of reducing equivalents and maximize the bioconversion of xylose into xylitol. The levels of glucose and xylose for co-fermentation experiments were optimized in shake flask followed by scale up of data in bioreactor. The impact of different feeding strategies on xylitol accumulation was examined. The batch and fed-batch mode of fermentations were conducted with pure xylose and the results were compared with crude xylose from SCB and OP.

## 2 Materials and methods

### 2.1 Preparation of LCB hydrolysates

The analytical grade chemicals purchased from Sigma Aldrich (USA), and Fischer Scientific (UK) were used in these investigations. The xylose-rich non-detoxified hemicellulosic hydrolysate obtained from hydrothermal pretreatment of SCB was kindly supplied by Nova Pangaea Technologies (https://www.novapangaea.com), Redcar, UK, our industrial partner. The SCB hydrolysate consisted of (g/L): 24.5, xylose; 2.4, arabinose; 2.7, glucose; 0.8, galactose; and 3.4, acetic acid. The crushed OP received from the olive mill (Unión Oleícola Cambil, Jaén, Spain) were treated with 2% w/v sulphuric acid solution using a solid loading of 50% w/v and incubated at 125 °C for 30 mins in an autoclave. After the slurry was brought at the room temperature, the solid residue was filtered to separate the liquid fraction consisting of hemicellulosic sugars. The liquid fraction was termed as OP hemicellulosic hydrolysate. The composition of OP hydrolysate was as follows (g/L): 84, xylose; 11, galactose; 4.7, glucose; 8.9, arabinose; 16.5, acetic acid; 1.2, formic acid; and 0.05, hydroxymethylfurfural (HMF). Both the hydrolysates were concentrated to xylose concentration of 400 g/L using vacuum distillation (Rotavapor, BUCHI UK Ltd), carried out overnight maintaining at 100 mbar, and 80 °C. For the media preparation during the fermentation experiments, the concentrated non-detoxified hydrolysates were diluted appropriately to meet the xylose concentrations.

### 2.2 Cultivation, inoculum preparation, and maintenance of microorganism

The xylose assimilating and xylitol accumulating yeast *P. fermentans* was isolated in our laboratory. The media for the culture maintenance and the pre-inoculum has been provided in our previous study (Prabhu et al., 2020a). The medium composition for the submerged fermentation was as follows (g/L): 18.36, yeast extract; 0.30, K_2_HPO_4_; 0.46, (NH_4_)_2_SO_4_; and 0.5, MgSO_4_. The xylose and glucose concentrations were adjusted according to the experimental requirements. The pre-inoculum was prepared by inoculating a colony of *P. fermentans* strain from a freshly sub-cultured plate into Erlenmeyer flask (100 mL) containing the YPX (10 g/L yeast extract; 20 g/L peptone; 20 g/L xylose) medium (50 mL) maintained at pH 7.0. The inoculated flasks were cultivated for 24 h in a rotary shaker at 30 °C with an agitation of 250 rpm.

### 2.3 Fermentation in shake flask

The submerged fermentation for xylitol accumulation were conducted in 500 mL Erlenmeyer flasks with 100 mL working volume. The inoculation was done using 24 h old pre-inoculum, and cultivated at 30 °C with an agitation rate of 250 rpm (Excella 24, New Brunswick). The co-fermentation experiments to determine optimal levels of glucose and xylose were performed with different ratios of pure glucose and xylose (10:90; 20:80; 30:70; 40:60; 50:50 w/w). Thereafter, the impact of various concentrations of pure xylose (50 – 200 g/L) with optimized glucose to xylose ratio on xylitol production was investigated. The effect of non-detoxified xylose-rich hydrolysates from SCB, and OP on substrate uptake, cell growth, and xylitol production were investigated. In case of non-detoxified hydrolysates, the glucose concentration was adjusted with addition of pure glucose to attain glucose to xylose ratio of 10:90.

### 2.4 Optimization of feed composition for improved xylitol production in a fed-batch mode of cultivation

The fed-batch fermentation for xylitol production was carried out in shake flasks using three different strategies to optimize the feed composition. In this context, three independent experiments were conducted with three different initial level of sugars and the culture was fed with xylose + glucose or xylose alone when initial xylose levels dropped to 5 – 10 g/L.

**Strategy I:** 50 g/L xylose with feeding of xylose alone.

**Strategy II:** 5 g/L glucose and 50 g/L xylose with feeding of mixture of glucose and xylose in ratio of 1:10.

**Strategy III:** 5 g/L glucose and 50 g/L xylose with feeding of xylose alone.

### 2.5 Fed-batch cultivation in a bioreactor with optimal feeding strategy

The fed-batch cultivation with optimal feeding strategy using pure sugars, non-detoxified SCB and OP hydrolysates were conducted in a bench-top bioreactor (2.5 L) (Electrolab, Bioreactors, UK) with 1.0 L working volume. The pre-inoculum and production media composition is as stated above. The temperature, agitation speed and aeration rate were maintained at 30 °C, 250 rpm and 1.0 vvm, respectively. The initial pH of the production media was adjusted to 7.0 prior to sterilization, and not controlled during the experiment.

### 2.6 Analytical techniques

The samples were collected at regular intervals to measure the microbial cell growth, residual glucose, xylose, and xylitol concentrations. The microbial cell growth was measured as optical density at 600 nm using a double beam spectrophotometer (Jenway 6310, UK). All the samples were appropriately diluted to bring OD_600_ in the range of 0.1–0.5. The cells from the fermented broth were separated by centrifugation at 10000 rpm for 10 min and the clear supernatant was permeated through 0.22 μm nylon membrane (Sartorious, Germany). The glucose, xylose, and xylitol concentrations were quantified using high performance liquid chromatography(Agilent Technologies 1200 series, USA), by eluting the filtered supernatant through Rezex ROA-organic acid H^+^ (Phenomenex, USA) column maintained at 60 °C, connected with a refractive index detector (RID) using 5.0 mM H_2_SO_4_ as mobile phase with flow rate of 0.4 mL/min. All the experiments conducted in this study were carried out in triplicates and the values provided are the average with standard deviation not >10%.

## 3 Results and discussion

### 3.1 Glucose and xylose co-fermentation by *P. Fermentans* in flask culture

**Fig. 1:**
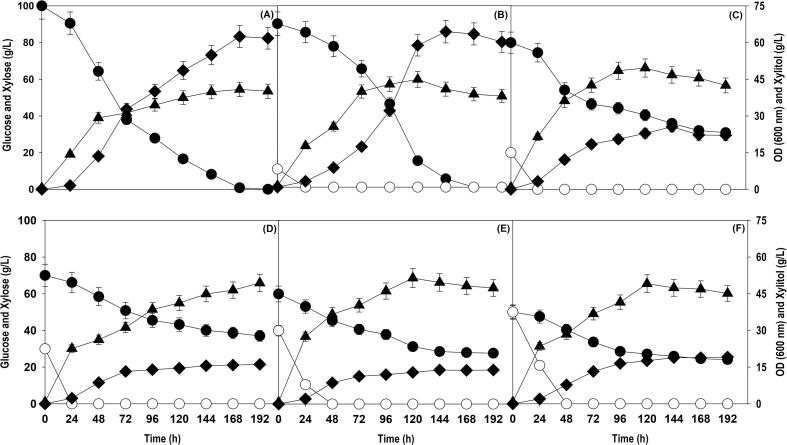
Shake flask cultivation of *P. fermentans* at different substrate concentrations (g/L): (A) 100 xylose (B) 10 glucose + 90 xylose; (C) 20 glucose + 80 xylose; (D) 30 glucose + 70 xylose; (E) 40 glucose + 60 xylose; and (F) 50 glucose + 50 xylose. Representations: filled circle (xylose); empty circle (glucose); filled triangle (OD_600_); filled diamond (xylitol). The graph represents the mean values from the triplicates with less than 10% standard deviation.

*P. fermentans* can grow and accumulate xylitol on xylose as sole carbon source and does not require a co-substrate for cell growth (Prabhu et al., 2020a) which is not the case with all xylitol accumulating cell factories. For example, *Yarrowia lipolytica* can transform xylose to xylitol, but cell mass of yeast cannot be grown on xylose as sole carbon source. Therefore, a co-substrate glycerol or glucose is needed for biomass formation and regeneration of co-factors [NAD(P)H] (Prabhu et al., 2020b). The other benefits of presence of a co-substrate are rapid build-up of active biomass and enhanced availability of redox co-factors [NAD(P)H] which can amplify the production parameters. In case of xylose fermenting yeast like *P. fermentans*, a significant fraction of xylitol produced is metabolized to generate biomass, reducing equivalents and maintenance energy which makes it challenging to achieve high xylitol yields. In many studies, a co-substrate has been added to avoid the dependence of strain on xylose for cell mass and/or co-factor supply (Kogje and Ghosalkar, 2017). Glucose is the most preferred carbon source for majority of the microorganisms therefore, it is envisaged that its addition will boost up the cell growth and eventually bioconversion of xylose to xylitol. To investigate the influence of glucose on xylose assimilation, cell growth and xylitol accumulation by *P. fermentans*, co-fermentation experiments were carried out with different glucose to xylose ratios (10:90; 20:80; 30:70; 40:60; 50:50 w/w). The profiles of xylose consumption, cell growth and xylitol production with respect to time are shown in Fig. 1. We observed that as the amount of glucose fraction was enhanced, there was a significant and continuous improvement in cell growth, however, the xylose utilised was not translated into xylitol. The high biomass formation indicates that desired path is not followed when cell growth dominates and is favoured at the expense of product formation. In fact, xylitol production and yield reduced with increase in glucose level. As a result of it, the fraction of residual xylose increased with increment in glucose concentration. The highest amount of xylitol was amassed with glucose to xylose ratio of 10:90. The supplied glucose (10 g/L) was depleted in 24 h followed by rapid xylose uptake with 100% utilization in 168 h. The maximum cell OD_600_ and xylitol titer obtained were 44.5 and 64.2 g/L, respectively, with xylose to xylitol conversion yield of 0.75 g/g. At other glucose to xylose ratio of 20:80, 30:70, 40:60, and 50:50, cell OD_600_ obtained were 49.6, 49.4, 51.5 and 49.1, however, the xylose was not fully utilized, and percent utilization was 61.3, 47.1, 53.8, and 51.8%, respectively. The same was reflected on the xylitol accumulation with maximum xylitol titer of 25.6, 16.1, 13.9 and 19.1 g/L with glucose to xylose ratio of 20:80, 30:70, 40:60, and 50:50, respectively. In case of control, without any glucose, the maximum cell OD_600_ of 40.8 along with xylitol titer and yield of 62.5 g/L and 0.63 g/g were attained. These results strongly suggests that fine tuning of glucose to xylose ratio is critical for amassing high xylitol levels. Since 1:10 was identified as optimal ratio and further experiments were carried out with a glucose to xylose ratio of 1:10.

The biosynthesis of xylitol is a reduction reaction catalysed by NAD(P)H dependent xylose reductase (XR) [xylose NAD(P)^+^ → xylitol + NAD(P)H]. After xylose enter the microbial cell, it is reduced to xylitol by XR. Some of the xylitol formed is secreted into extracellular medium and rest is oxidized into xylulose with NAD^+^ as electron acceptor and the reaction is catalysed by xylitol dehydrogenase (XDH). Xylulose formed subsequently enter central carbon metabolism for biosynthesis of precursors and eventually contributes to microbial growth (Tiwari and Baghela, 2020, Xu et al., 2019). The favourable conditions for the improved accumulation of xylitol are the continuous supply of reducing equivalents and secretion of it without being further metabolized (Bengtsson et al., 2009). It has been hypothesized that the presence of glucose enhances the supply of NADPH and biomass formation mainly through pentose phosphate pathway. Theoretically, twelve moles of NADPH can be generated from every mole of glucose consumed. The presence of glucose in the growth or fermentation medium ensures continuous supply of NADPH to drive smooth bioconversion of xylose into xylitol by XR. As a result of it, a small quantity of xylitol is funnelled into cell metabolism, and more is pumped out into extracellular environment (Tochampa et al., 2005, Wannawilai et al., 2017). Glucose/xylose ratios > 10% have been reported to negatively impact the transport of xylose and inhibit the action of XR and a ratio less than or equal to 10% could enhance the enzymatic action (López-Linares et al., 2020, Tochampa et al., 2005).

Our results are in agreement with previous reports where it has been suggested that a glucose to xylose ratio of 1:10 is optimal for xylitol accumulation. Wannawilai and associates found that 1:10 mass ratio of glucose to xylose stimulated volumetric and specific rates of xylose consumption and xylitol production by *Candida magnoliae* TISTR 5663*,* while significant drop (30–35%) in specific and volumetric production rate was observed in absence of glucose (Wannawilai et al., 2017). López-Linares et al. (2018) investigated xylitol production by *Debaryomyces hansenii* NRRL Y-7426 and *Candida guilliermondii* ATCC 201,935 and noticed that higher glucose/xylose ratio (>30%) present in rapeseed straw hemicellulosic hydrolysate and semi-defined media retarded the bioconversion process. Oh and Kim (1998) performed xylose + glucose co-fermentation by *Candida tropicalis* KFCC-10960 with different levels of glucose. The maximum xylitol production was achieved with a xylose and glucose concentration of 100 and 10 g/L, respectively. Further increase in the glucose concentration beyond 10 g/L, the metabolism was shifted towards ethanol production causing reduction in xylitol yield. Similarly with studies of Tavares et al., 2000, the glucose to xylose ratio of 1:10 resulted in xylitol yield and productivity of 0.56 g/g and 0.21 g/L/h, respectively, which was 30% higher than using xylose as sole carbon source. The higher glucose/xylose ratios inhibit xylitol production through impeding xylose transport and repression of relevant enzymes. It is a known fact that, glucose is the preferred carbon source over xylose, and compete with the uptake of xylose but does not yield xylitol. In fact, the xylose assimilation is inhibited in the presence of glucose due to carbon catabolite repression or glucose effect unlike glycerol which could be co-metabolized with xylose (Bruder et al., 2015, Zhang et al., 2015). Therefore, if glucose is present in excess, it will overtake xylose metabolism and severely impact xylitol formation. Contrary with most of the reports, Zhang and associates reported a glucose to xylose ratio of 2:1 for optimal xylitol manufacturing by engineered *Kluyveromyces marxianus* overexpressing galactose permease and XR with deleted XDH gene. The engineered strain accumulated 140 g/L xylitol through co-fermentation of 70 g/L glucose and 140 g/L xylose with a productivity of 0.83 g/L/h. Due to absence of XDH, xylose supplemented was biotransformed into xylitol, and glucose was used as carbon source for growth and development (Zhang et al., 2021).

### 3.2 Impact of enhancing xylose level on xylitol accumulation from co-fermentation

**Fig. 2:**
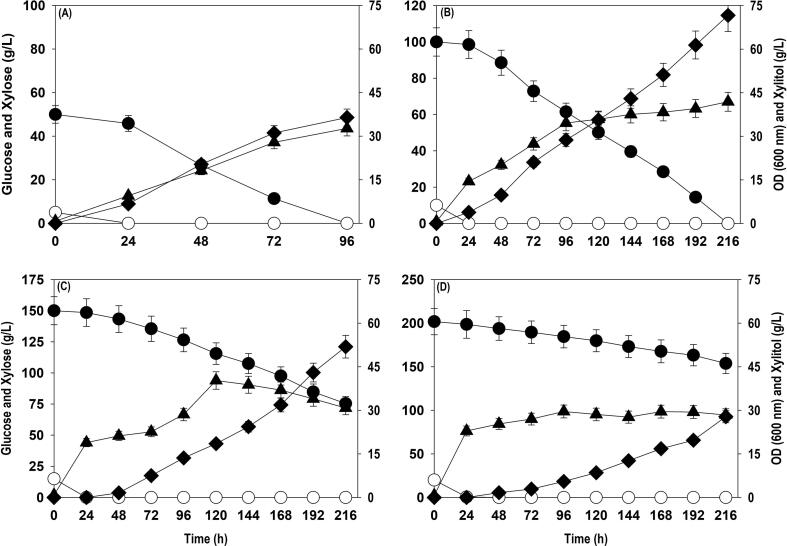
Co-fermentation of different concentrations (g/L) of glucose and xylose by *P. fermentans* at 1:10 glucose to xylose ratio: (A) 5 glucose + 50 xylose; (B) 10 glucose + 100 xylose; (C) 15 glucose + 150 xylose; (D) 20 glucose + 200 xylose. Representations: filled circle (xylose); empty circle (glucose); filled triangle (OD_600_); filled diamond (xylitol). The graph represents the mean values from the triplicates with less than 10% standard deviation.

After determining the optimal ratio, the shake flask experiments were conducted with different concentrations of xylose (50, 100, 150 & 200 g/L) with glucose to xylose ratio of 1:10 to understand the limits of xylose consumption in a batch culture, identify the optimal level and limit beyond which substrate inhibition comes into the picture and how it impacts cell growth, and xylitol production by *P. fermentans*. Fig. 2 depicts time course profiles of xylose and glucose uptake, cell growth (OD_600_) and xylitol accumulation. In all the cases 100% glucose consumption was detected within 24 h, while xylose was completely exhausted with initial xylose concentrations of 50 and 100 g/L only. The xylitol titer quantified at the end of fermentation with 50 and 100 g/L were 36.4 and 71.6 g/L xylitol with a yield of 0.73 and 0.72 g/g, respectively. Cultures with 50 and 100 g/L xylose did not exhibit any lag phase and an active biomass production from the beginning was noticed while at higher substrate concentrations (150 and 200 g/L) distinct lag phase and substrate inhibition effects were observed. The increase in xylose concentrations to 150 and 200 g/L reduced the xylose consumption efficiency and the fraction of supplied xylose left unconsumed at the end of fermentation were 50.3 and 76.1%, respectively. Accordingly, xylitol production was negatively affected and the xylitol titer obtained with 150 and 200 g/L xylose were 51.9 and 27.8 g/L with conversion yield of 0.70 and 0.58 g/g, respectively.

In literature *Candida* sp.*,* have been the most investigated cell factories for xylitol production. Zhang et al. (2012) evaluated the effect of initial xylose concentrations (100–300 g/L) on xylitol production by a new yeast isolate *Candida athensensis* SB18. The substrate inhibition was noticed at higher xylose levels. The maximum xylitol accumulation of 162.7 g/L with yield and productivity of 0.82 g/g and 0.85 g/L/h, respectively, was observed at an initial xylose concentration of 250 g/L with a significant amount of residual xylose (51.7 g/L). Further increase in xylose level to 300 g/L decreased the substrate consumption with large amount of unutilized xylose (107.6 g/L) and also caused reduction in xylitol titer (146.7 g/L), yield (0.76 g/g) and productivity (0.64 g/L/h). Similarly, in a study reported by Oh and Kim, 1998, enhanced xylitol accumulation (38.2 – 225 g/L) by *Candida tropicalis* KFCC-10960 was observed with an increment in initial xylose concentrations (50 – 250 g/L), but biomass and xylitol formation rate dropped beyond 150 g/L xylose. Similar to our results, Kim et al., (2002) reported 100 g/L as optimal xylose level for biosynthesis of xylitol by *C. tropicalis* ATCC 13,803 in batch fermentation and further increase in the initial xylose concentrations, hindered the cell growth and xylitol productivity. Ping et al., (2013) evaluated the capability of *C. tropicalis* CCTCC M2012462 to manufacture xylitol at different concentration of pure and crude xylose. In case of pure xylose, as the substrate was elevated from 30 to 60 g/L, there was a continuous and steady increment in xylitol yield with maximum of 0.71 g/g at 60 g/L xylose. The cell growth and productivity were higher in pure xylose when compared with hydrolysates. The xylitol yield (0.72 g/g) was highest at 40 g/L xylose in hydrolysate. The fermentation time was prolonged as cell took time to completely degrade furfural and 5-HMF. Further enhancement in xylose level (60 g/L) caused a severe decline in cell growth and xylitol productivity as amount of inhibitors also increased with xylose concentration. Though *Candida* sp. has resulted in high level accumulation of xylitol using pure sugars or hemicellulosic hydrolysates, they are opportunistic pathogens which impede their industrial application. On the contrary, *P. fermentans* is a non-pathogenic yeast which is known to produce flavour and aroma compounds like 2-phenyl ethanol (Chung et al., 2000). Along with these characteristics, the yeast posses’ antagonistic properties that prevent the bacterial and fungal contamination and further enhance the shelf-life of food and beverage products (Pal and Sharma, 2019). *P. fermentans* can be the better chassis strain to be looked forward, as xylitol has major applications in food, medical, and pharmaceutical industries.

### 3.3 Xylitol production from non-detoxified SCB and OP hydrolysates in shake flask

**Fig. 3:**
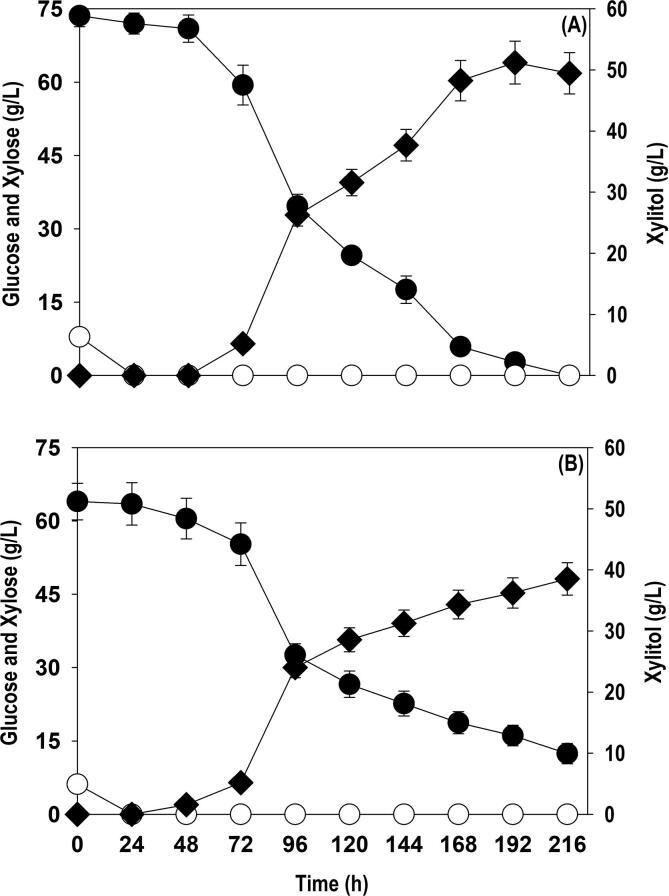
Kinetics of xylose utilization and xylitol accumulation by *P. fermentans* using xylose rich hemicellulosic hydrolysates from: (A) SCB; (B) OP. Representations: filled circle (xylose); empty circle (glucose); filled diamond (xylitol). The graph represents the mean values from the triplicates with less than 10% standard deviation.

SCB is major solid waste stream generated from sugar mills manufacturing table sugar from sugarcane crop. The global cultivation of sugarcane crop was 1841 million tonnes (Mt) in 2017 with Brazil (756 Mt), India (304 Mt), China (104 Mt) and Thailand (103 Mt) as the major contributors. SCB is the largest agriculture residue in the world and crushing of one tonne of sugarcane generates ∼ 0.3 tonne of SCB leading to annual global production of ∼ 550 Mt. Currently, SCB is inefficiently burnt in boiler to provide heat in sugar mills (Konde et al., 2021, Meghana and Shastri, 2020). In 2018, around 10.7 million hectares of land is under olive cultivation in 41 different countries around the globe, with a total production of 21.6 million tonnes of olives generating 3.2 million tonnes of oil. European countries like Spain and Italy are the major producers. The OP (8 – 12% w/w olives) is the solid or stones removed from the olive pomace (50 – 60% w/w olives), a by-product of olive cultivation and oil extraction. Various other by-products like olive tree pruning (1.5 tonne/hectare), and olive leaves (5 – 10% w/w olives) rich in carbohydrate content are an attractive feedstock for biorefinery (Contreras et al., 2020). The xylose-rich hydrolysate was obtained from SCB and OP via acid hydrolysis. The composition of the non-detoxified SCB and OP hydrolysates is provided in the section 2.1. The xylose-rich hydrolysate from SCB and OP was used for manufacturing of xylitol through co-fermentation and time course profiles for substrate assimilation and xylitol formation are shown in Fig. 3. The hydrolysates were translucent and consisted of particles even after sedimentation which could interfere with OD measurement, therefore cell growth was not quantified. The glucose was quickly consumed with in 24 h in both the cases. The xylitol production commenced from 72 and 48 h in the reactor supplemented with SCB and OP hydrolysates respectively, which was a delayed response in comparison to pure xylose where it started within 24 h, after depletion of glucose and overall xylitol production rate was substantially slower. The SCB-derived xylose was completely exhausted in 216 h with xylitol accumulation of 49.5 g/L and yield of 0.67 g/g. In comparison to SCB, ∼80.6% xylose was consumed in case of OP resulting in xylitol titer and yield of 38.5 g/L and 0.75 g/g, respectively. The difference in the product titer and yield could be attributed to presence of higher acetic acid concentration in OP hydrolysate, and during the pretreatment of LCB residues, various metal ions are leached out, that might interfere with the cell growth and fermentation efficiency.

As mentioned above that *P. fermentans* was isolated in our laboratory and there is no information in literature about its capability to manufacture xylitol. In our previous work, the *P. fermentans* was cultured in shake flask for xylitol production from pure xylose and SCB derived xylose-rich non-detoxified hydrolysate as carbon sources with initial xylose concentration adjusted to 150 g/L. The yeast strain was able to produce 70.5 and 62.3 g/L xylitol with conversion yield of 0.49 and 0.43 g/g from pure xylose and SCB hydrolysate, respectively (Prabhu et al. 2020a). In the current study, the xylitol yield obtained on pure xylose, SCB and OP hydrolysate are significantly higher than the previous work indicating the benefit of presence of glucose at small levels (∼10% of xylose concentration). There is substantial literature available on SCB-based xylitol accumulation but scarce on xylitol production using waste streams from olive industries. Recently, López-Linares et al. (2020) evaluated xylitol production from hemicellulosic hydrolysate of exhausted olive pomace (EOP) by *Candida boidinii* NCAIM Y.01308. EOP is obtained as a solid residue after extraction of the remaining oil present in the olive pomace with hexane and contains cellulose, hemicellulose, lignin, and extractives. The yeast was not able to manufacture xylitol from EOP hydrolysate without detoxification which could be due to presence of inhibitors mainly furans, acetic acid, and phenolic compounds. Further the hydrolysate was detoxified using two techniques, activated carbon (AC) and ion-exchange resin (IER). The xylitol concentration achieved from AC and IER treated hydrolysates were 6.0 and 5.2 g/L with yield of 0.43 and 0.36 g/g, respectively. They suggested that besides fermentation inhibitors, xylitol production is also negatively influenced by considerable presence of glucose with glucose to xylose ratio > 10 in hydrolysates. Comparison with our results clearly demonstrate the potential of *P. fermentans* which produced much higher level of xylitol from ORP and that too on a non-detoxified hydrolysate.

### 3.4 Fed-batch culture in shake flask with different feeding strategies

**Fig. 4:**
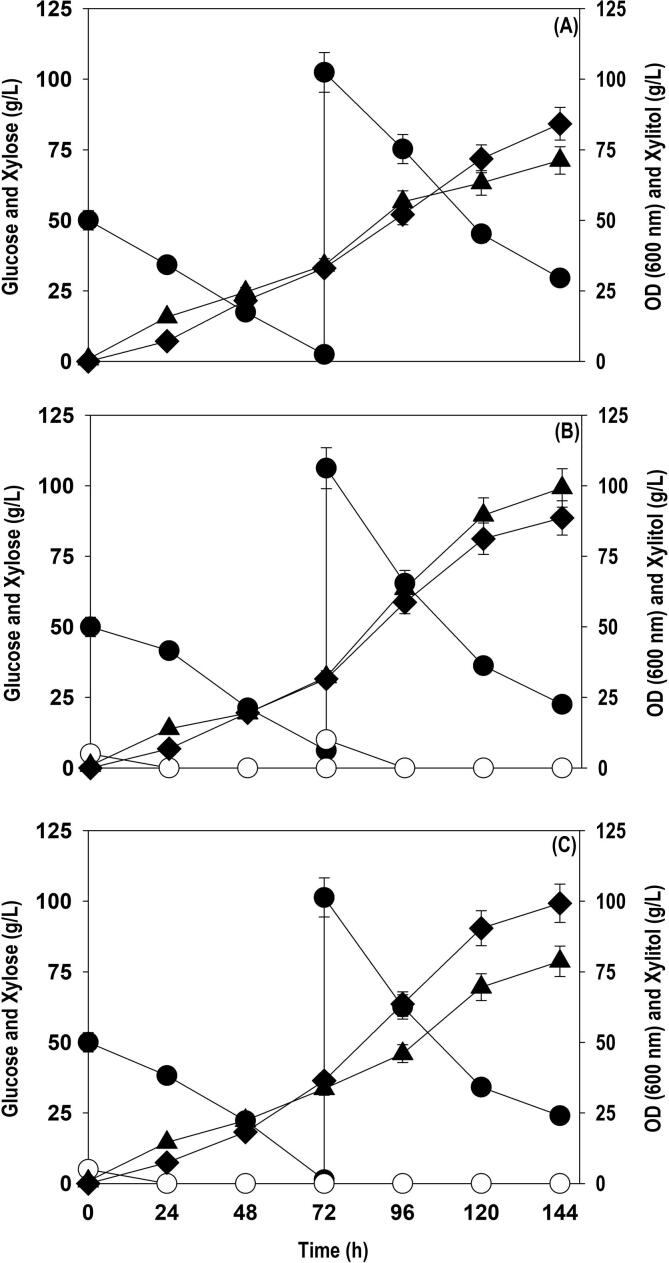
Effect of different feeding strategies on xylose assimilation, cell growth (OD_600_) and xylitol accumulation during fed-batch culture in shake flasks: (A) Strategy I; (B) Strategy II; (C) Strategy III. Representations: filled circle (xylose); empty circle (glucose); filled triangle (OD_600_); filled diamond (xylitol). The graph represents the mean values from the triplicates with less than 10% standard deviation.

To improve the final titers of xylitol, the fed-batch fermentation with different feeding strategy was performed in shake flask cultivations. The variation in glucose and xylose consumption, cell growth and xylitol accumulation with different feeding strategies as a function of fermentation time is shown in Fig. 4. In case of strategy I, the batch cultivation was started with initial working volume of 75 mL in 500 mL Erlenmeyer flask. The batch cultivation yielded a cell OD_600_ of 34.1 and xylitol accumulation of 33.1 g/L xylitol at 72 h when the culture was fed with 100 g/L xylose. Further addition of xylose improved cell growth and xylitol level. The OD_600_ and xylitol accumulated at 144 h were 71.2 and 84.2 g/L with overall yield of 0.70 g/g. However, all the supplied xylose was not assimilated and residual xylose at 144 h was 29.5 g/L. In strategy II, fermentation was initiated with 5 g/L glucose + 50 g/L xylose. The batch reaction progressed until 72 h where cell growth (OD_600_) and xylitol titer attained were 32.2 and 31.6 g/L, respectively. The feeding with 10 g/L glucose and 100 g/L xylose was done at 72 h when initial xylose level dropped to less than 5.0 g/L. The feeding accelerated the biomass and xylitol formation rate. As a result of it, the cell OD_600_ and xylitol titer improved from 32.2 to 99.2 and 31.6 to 88.6 g/L, respectively, at 144 h and residual xylose of 22.5 g/L was observed at the end. The xylitol conversion yield was 0.70 g/g similar to strategy I. In strategy III, the initial sugar levels (5 g/L glucose + 50 g/L xylose) were same as that of strategy II. The pattern of batch fermentation observed was similar to strategy II and comparable xylitol concentration (36.4 g/L) and cell growth (OD_600_: 33.5) was obtained. Feeding with xylose at 100 g/L caused significant enhancement in xylitol formation in next 48 h and thereafter, a slow and steady increase was observed. The xylitol accumulated was 99.3 g/L with conversion yield of 0.80, though all the fed xylose was not metabolized. The xylitol titer and yield recorded with strategy III was highest among the three strategies employed. It was evident that the presence of glucose in strategy II and III largely facilitated cell growth in comparison to strategy I with no glucose. The results also shows that presence of glucose in the fermentation medium is beneficial for boosting up the xylitol accumulation. However, feeding xylose along with glucose remarkably enhanced the biomass formation but it did not positively influence the manufacturing of xylitol by the yeast. The fed-batch experiments in 2.5L bioreactor using pure sugars, SCB and OP hydrolysates were conducted using strategy III.

### 3.5 Fed-batch cultivation in a bioreactor with optimal feeding strategy

**Fig. 5:**
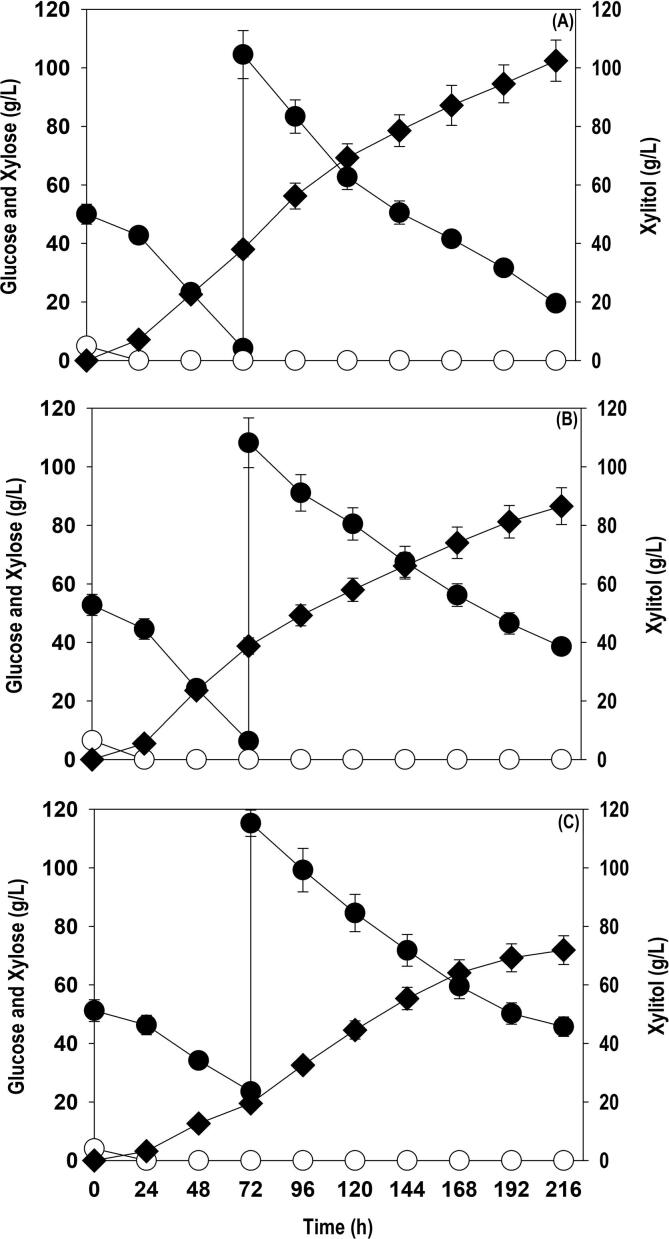
Time course profiles of xylose assimilation, cell growth (OD_600_) and xylitol accumulation during fed-batch cultivation of *P. fermentans* in bioreactor on: (A) pure xylose; (B) non-detoxified SCB; and (C) non-detoxified OP hydrolysates. Representations: filled circle (xylose); empty circle (glucose); filled diamond (xylitol). The graph represents the mean values from the triplicates with less than 10% standard deviation.

As observed in the shake flask experiments, with initial 5 g/L glucose + 50 g/L xylose, and further feeding with xylose alone displayed the highest bioconversion of xylose to xylitol. The similar feeding strategy using pure sugar, SCB and OP hydrolysates were replicated in bioreactor with feeding of 100 g/L xylose at 72 h. The media components and the culture conditions were similar to the shake flask experiments except aeration where air at flow rate of 1.0 L/min was provided. Fig. 5 depicts the profiles of xylose feeding, consumption rate, cell growth and xylitol accumulation by *P. fermentans* strain using pure xylose (Fig. 5A), SCB (Fig. 5B), and OP (Fig. 5C) hydrolysates. The pattern of growth, substrate consumption observed in the bioreactor was similar to shake flask experiments. The glucose supplemented (5–7 g/L) in the beginning was consumed in less than 24 h. As we discussed above that the presence of glucose in batch phase significantly improved biomass production which eventually enhanced the xylose uptake rate in subsequent phases. As a result of it, 80–100 g/L xylose was assimilated in 48–144 h. The maximum xylitol produced from pure xylose, SCB and OP hydrolysate were 102.5, 86.6 and 71.9 g/L at 216 h with conversion yield of 0.78, 0.75 and 0.74 g/g, respectively. The increased titer and yield of xylitol in the bioreactors compared to the shake flask cultivation could be due to improved mass transfer caused by sufficient aeration and better mixing. The decline in xylitol productivity in later stages of fermentation may be due to end-product toxicity. Xylitol being a sugar alcohol at high concentration causes high oxidative stress and affect cell membrane fluidity resulting in reduced xylose assimilation rate (Kim et al., 2002). The difference in the titers and yield between the pure sugars and the hydrolysates may be due to presence of fermentation inhibitors like acetic acid, metal ions, lignin derivatives and other undetected ions or chemicals (Ping et al., 2013, López-Linares et al., 2020).

Bacteria, yeasts and fungi have opened the possibility to synthesize xylitol using xylose present in a large number of crude renewable sources. SCB and corncob due to their abundance and good hemicellulosic content have been majorly utilized feedstock for bioproduction of xylose-based metabolites including xylitol. Vaz de Arruda et al., 2017 performed xylitol fermentation by *Candida guilliermondii* FTI 20,037 using detoxified SCB hydrolysate and data was scaled up. However, xylitol titer and yield dropped from 36.6 to 20.8 g/L and 0.65 to 0.55 g/g when the data was scaled up from 2.4 to 125 L bioreactor. Vallejos et al., (2016) detoxified the SCB hydrolysate through a sequence of treatments including Ca(OH)_2_, IR-120 resin, activated charcoal, and IRA-67 resin which removed all inhibitors from the hemicellulosic hydrolysates. The fermentation of inhibitor free hydrolysate by *C. tropicalis* yielded 32 g/L xylitol with yield of 0.46 g/g. We would like to mention two reports making use of non-detoxified and detoxified hemicellulosic corncob hydrolysate for xylitol production. Misra et al. (2013) accumulated 11.9 g/L xylitol with a volumetric yield of 0.58 g/g by an adapted strain of *C. tropicalis* using detoxified corncob hydrolysate. Despite adaptation of strain, the production was not high. On the other hand, Ping et al. (2013) performed fed-batch cultivation using non-detoxified hydrolysate with low inhibitor concentration. The strain *C. tropicalis* CCTCC M2012462 was able to amass 38.8 g/L after 84 h with product yield of 0.70 g/g. In another study, Cheng and associates cultivated the strain *C. tropicalis* W103 on the detoxified corncob hydrolysate, accumulating 68.4 g/L xylitol with a conversion yield and productivity of 0.70 g/g, and 0.95 g/L/h, respectively (Cheng et al., 2009). Cheng and associates also found that the presence of glucose in the hydrolysate improved the cell growth and xylitol production.

In addition to SCB and corncob, various other lignocellulosic feedstocks have been examined for biosynthesis of xylitol. Ko and associates made use of detoxified and concentrated hydrolysate from hardwood waste for xylitol production by various *Candida* strains. The maximum xylitol concentration with *Candida boidinii* (BCRC 21432), *C. guilliermondii* (BCRC 21549), *C. utilis* (BCRC 20334), *P. anomala* and *C. tropicalis* (BCRC 20520) were 6.1, 40.7, 12.5, 36.0 and 41.4 g/L with a volumetric yield of 0.11, 0.69, 0.21, 0.60 and 0.70 g/g, respectively (Ko et al., 2008). All these yeast strains also co-produced a significant amount of ethanol. Kim, 2019 cultivated the *Candida tropicalis* CBS94 strain on detoxified xylose-rich hemicellulosic hydrolysate obtained from acid pretreatment of empty palm fruit bunches, resulting in xylitol titers of 35.2 g/L with a conversion yield and productivity of 0.44 g/g and 0.58 g/L/h, respectively (Kim, 2019). Santana et al., (2018) employed cocoa pod husk, waste from cocoa cultivation, for xylitol production by *C. boidinii* XM02G. The fermentation of detoxified cocoa pod husks hemicellulosic hydrolysate by *C. boidinii* XM02G resulted in xylitol concentration of 11.3 g/L after 372 h with a yield of 0.52 g/g. The hydrolysate for this purpose was detoxified through pH adjustment and activated charcoal treatment. Detoxification for removal of desired molecules not only decrease the available sugar concentrations, but also raise the operational cost. As we have described above that only few studies have focused on the fermenting biomass hydrolysate without detoxification. Even without detoxification, the xylose assimilation rate, cell growth, and xylitol production quantified in this study are analogous or much better than many of the reports discussed. The performance of *P. fermentans* strain on detoxified SCB and OP hemicellulosic hydrolysate in terms of xylose uptake and xylitol accumulation were comparable to pure sugars, indicating the capability and efficiency of this non-conventional yeast as a chassis strain for valorisation of hemicellulosic hydrolysates. The co-substrate-based xylitol fermentation by *P. fermentans* caused significant improvement in product yield (pure xylose: 0.78 vs 0.67 g/g; SCB hydrolysate: 0.75 vs 0.54 g/g) in comparison to our previous work (Prabhu et al. 2020a) where xylitol was accumulated on commercial xylose and SCB hydrolysate without any exogenous addition of glucose. This is really advantageous for 2G feedstocks where small level of glucose is already there in hydrolysate.

## 4 Conclusion

The current study investigated xylitol production from two major agro-industrial wastes, SCB and OP by *P. fermentans*. The co-fermentation of xylose and glucose resulted in high xylitol titers using pure sugars, SCB and OP hydrolysates. Fine-tuning of the glucose to xylose ratio was identified to be critical as glucose concentrations > 10 g/L boosted the biomass build up but negatively impacted the xylitol accumulation. The efficient valorisation of overlooked hemicellulosic fraction into valuable product like xylitol will significantly contribute to profitability of lignocellulosic biorefineries. Future research could focus on strain and process engineering strategies to meet the requirements for industrial manufacturing of xylitol.

## CRediT authorship contribution statement

**Vivek Narisetty:** Conceptualization, Methodology, Software. **Eulogio Castro:** Methodology. **Sumit Durgapal:** Conceptualization. **Frederic Coulon:** Data curation. **Samuel Jacob:** Methodology. **Dinesh Kumar:** Methodology. **Mukesh Kumar Awasthi:** . **Kamal Kishore Pant:** . **Binod Parameswaran:** . **Vinod Kumar:** .

## Declaration of Competing Interest

The authors declare that they have no known competing financial interests or personal relationships that could have appeared to influence the work reported in this paper.
